# Overview of BioCreative II gene mention recognition

**DOI:** 10.1186/gb-2008-9-s2-s2

**Published:** 2008-09-01

**Authors:** Larry Smith, Lorraine K Tanabe, Rie Johnson nee Ando, Cheng-Ju Kuo, I-Fang Chung, Chun-Nan Hsu, Yu-Shi Lin, Roman Klinger, Christoph M Friedrich, Kuzman Ganchev, Manabu Torii, Hongfang Liu, Barry Haddow, Craig A Struble, Richard J Povinelli, Andreas Vlachos, William A Baumgartner, Lawrence Hunter, Bob Carpenter, Richard Tzong-Han Tsai, Hong-Jie Dai, Feng Liu, Yifei Chen, Chengjie Sun, Sophia Katrenko, Pieter Adriaans, Christian Blaschke, Rafael Torres, Mariana Neves, Preslav Nakov, Anna Divoli, Manuel Maña-López, Jacinto Mata, W John Wilbur

**Affiliations:** 1 National Center for Biotechnology Information, Bethesda, Maryland, USA; 2 IBM TJ Watson Research Center, Yorktown Heights, NY, USA; 3 Institute of Bioinformatics, National Yang-Ming University, Taipei, Taiwan; 4 Institute of Information Science, Academia Sinica, Taipei, Taiwan; 5 Fraunhofer Institute for Algorithms and Scientific Computing (SCAI), Department of Bioinformatics, Schloss Birlinghoven, Sankt Augustin, Germany; 6 Department of Computer and Information Science, University of Pennsylvania, Philadelphia, Pennsylvania, USA; 7 Department of Biostatistics, Bioinformatics and Biomathematics, Georgetown University Medical Center, Washington, District of Columbia, USA; 8 School of Informatics, University of Edinburgh, UK; 9 Department of Mathematics, Statistics and Computer Science, Marquette University, Milwaukee, Wisconsin, USA; 10 Department of Electrical and Computer Engineering, Marquette University, Milwaukee, Wisconsin, USA; 11 Computer Laboratory, University of Cambridge, Cambridge, UK; 12 University of Colorado School of Medicine, Center for Computational Pharmacology, Denver, Colorado, USA; 13 Alias-i, Inc., Brooklyn, New York, USA; 14 Institute of Information Science, Academia Sinica, Taipei, Taiwan; 15 Department of Computer Science & Engineering, Yuan Ze University, Taoyuan City, Taiwan; 16 Department of Computer Science, National Tsing-Hua University, Hsinchu, Taiwan; 17 Computational Modeling Laboratory, Vrije Universiteit Brussels, Belgium; 18 School of Computer Science and Technology, Harbin Institute of Technology, Harbin, China; 19 Human Computer Studies Laboratory, Institute of Informatics, University of Amsterdam, Amsterdam, The Netherlands; 20 Bioalma, Tres Cantos (Madrid), Spain; 21 Facultad de Informática, Universidad Complutense de Madrid, Madrid, Spain; 22 Department of Electrical Engineering and Computer Sciences, Computer Science Division, University of California, Berkeley, California, USA; 23 Bulgarian Academy of Sciences, Institute for Parallel Processing, Linguistic Modeling Department, Sofia, Bulgaria; 24 School of Information, University of California, Berkeley, California, USA; 25 Departamento de Tecnologías de la Información, Universidad de Huelva, Huelva, Spain

## Abstract

Nineteen teams presented results for the Gene Mention Task at the BioCreative II Workshop. In this task participants designed systems to identify substrings in sentences corresponding to gene name mentions. A variety of different methods were used and the results varied with a highest achieved F_1 _score of 0.8721. Here we present brief descriptions of all the methods used and a statistical analysis of the results. We also demonstrate that, by combining the results from all submissions, an F score of 0.9066 is feasible, and furthermore that the best result makes use of the lowest scoring submissions.

## Background

Finding gene names in scientific text is both important and difficult. It is important because it is needed for tasks such as document retrieval, information extraction, summarization, and automated text mining, reasoning, and discovery. Technically, finding gene names in text is a kind of named entity recognition (NER) similar to the tasks of finding person names and company names in newspaper text [1]. However, a combination of characteristics, some of which are common to other domains, makes gene names particularly difficult to recognize automatically.

• Millions of gene names are used.

• New names are created continuously.

• Authors usually do not use proposed standardized names, which means that the name used depends on preference.

• Gene names naturally co-occur with other types, such as cell names, that have similar morphology, and even similar context.

• Expert readers may disagree on which parts of text correspond to a gene name.

• Unlike companies and individuals, genes are not defined unambiguously. A gene name may refer to a specified sequence of DNA base pairs, but that sequence may vary in nonspecific ways, as a result of polymorphism, multiple alleles, translocation, and cross-species analogs.

All of these things make gene name finding a unique and persistent problem. An alternative approach to finding gene names in text is to decide upon the actual gene database identifiers that are referenced in a sentence. This is the goal of the gene normalization task [2]. While success in gene normalization to some degree eliminates the need to find explicit gene mentions, it will probably never be the case that gene normalization is more easily achieved. Therefore, the need to find gene mentions will probably continue into the future.

### Task description

BioCreative is a 'challenge evaluation' (competition or contest), in which participants are given well defined text-mining or information extraction tasks in the biological domain. Participants are given a common training corpus, and a period of time to develop systems to carry out the task. At a specified time the participants are then given a test corpus, previously unseen, and a short period of time in which to apply their systems and return the results to the organizers for evaluation. All submissions are then evaluated according to numerical criteria, specified in advance. The results are then returned to the participants and subsequently made public in a workshop and coordinated publication. The first BioCreative challenge was carried out in 2003 (with a workshop in 2004) and consisted of a gene mention task, a gene normalization task and a functional annotation task. The current BioCreative challenge took place in 2006 and the workshop in April of 2007. There were three tasks in 'BioCreative II', called the gene mention (GM), gene normalization (GN) and protein-protein interaction (PPI) tasks.

The BioCreative II GM task builds on the similar task from BioCreative I [3]. The training corpus for the current task consists mainly of the training and testing corpora (text collections) from the previous task, and the testing corpus for the current task consists of an additional 5,000 sentences that were held 'in reserve' from the previous task. In the time since the previous challenge, the corpus was reviewed for consistency using a combined automated and manual process. In the previous task, participants were asked to identify gene mentions by giving a range of tokens in the pretokenized sentences of the corpus. In the current corpus, tokenization is not provided; instead participants are asked to identify a gene mention in a sentence by giving its start and end characters. As before, the training set consists of a set of sentences, and for each sentence a set of gene mentions (GENE annotations). Each 'official' GENE annotation in a sentence may optionally have alternate boundaries that are judged by human annotators to be essentially equivalent references (ALTGENE annotations).

Every string identified by a run is considered either a true positive or a false positive. If the string matches a GENE or ALTGENE in the humanly annotated corpus, it is counted as a true positive with the exception that only one true positive is permitted per gene given in the corpus. If none of the annotations of a gene given in the corpus match a string nominated by a run, then the gene is counted as a false negative. A run is scored by counting the true positives (*TP*), false positives (*FP*), and false negatives (*FN*). Let *T *= *TP *+ *FN *denote the total number of genes in the corpus, and let *P *= *TP *+ *FP *denote the total number of nominated gene mentions by a run. The evaluation is based on the performance measures *p *(precision), *r *(recall), and their harmonic average *F*:

$$\begin{array}{c} p=TP/P\\ r=TP/T\\ F={\left(\frac{p^{-1}+r^{-1}}{2}\right)}^{-1}=TP/(T+P)/2. \end{array}$$

Different applications may favor a different weighting between precision and recall, but this is beyond the scope of our analysis. We assume this simple form of F score in all of our analysis.

Despite being called a 'challenge evaluation', competition, or contest, there are several reasons to view the results differently. As is pointed out repeatedly in the TREC workshop [4], the 'absolute value of effectiveness measure is not meaningful', that is, the scores provided are not meaningful outside of the context of the challenge. The F score is a specific metric, not without controversy, and the value achieved on the corpora of the challenge is no guarantee of performance on other corpora. We demonstrated in [5] how it may be possible to estimate the performance on alternative corpora. All performance measures have a natural statistical variation, even within the narrow confines of the corpora defined for this task. We have estimated the statistical significance of pair-wise comparisons. Finally, runs that score below the median may still give valuable insights into the task, and we have provided some evidence that this is the case. In short, this competition is not a horse race, but a scientific forum in which the state-of-the-art is advanced through comparison and sharing of ideas.

### Corpus preparation

In 2003, as part of a project to improve on the AbGene tagger [6], a corpus of 20,000 sentences was selected and annotated for training and testing purposes [7]. As described in [6], a Bayesian classifier was developed to recognize documents that are likely to contain gene names, and it was found that the precision and recall of the tagger was much better for high scoring documents. With this motivation, 10,000 sentences from high scoring documents and 10,000 sentences from low scoring documents were selected and combined to form the 20,000 sentence corpus. The corpus was further subdivided into train, test, round1, and round2 sets of 5,000 sentences, each of which contained equal numbers of high scoring and low scoring sentences. The train and test sets were provided as the training set in BioCreative I, and the round1 set was used as the final evaluation. With some modifications, the train, test, and round1 sets were provided as the training set in BioCreative II, and the round2 set was used as the final evaluation.

For BioCreative II, the entire corpus of 20,000 sentences and approximately 44,500 GENE and ALTGENE annotations, was converted to the MedTag database format [8]. To do this, the original sentence in Medline^® ^was located (although a few had been removed from Medline and were replaced with sentences existing at the time). The bibliographic information for each sentence was also determined. The token specifications of all previous annotations were changed to character specifications. Also, because annotations were no longer limited to preset token boundaries, it was necessary to review manually every annotation to confirm or relocate the annotation boundaries. For example, it became possible to annotate a gene that is hyphenated to another word, the combination of which is not a gene mention.

To improve the consistency of annotation, approximately 1,500 strings (containing two or more characters) were found that were annotated as GENE or ALTGENE in one sentence and unannotated in another sentence. These strings occurred in approximately 13,500 mentions, of which 4,300 were GENE annotations, 2,200 were ALTGENE annotations, and 7,000 were unannotated. All of these cases were manually reviewed for accuracy and corrections were made. Overall, 13.9% of the sentences had changes in GENE annotations and 13.0% of the sentences had changes in ALTGENE annotations over the period of time between BioCreative I and BioCreative II.

## Results and discussion

The BioCreative I gene mention task had 15 participants and each was allowed to submit up to four runs, categorized as either closed (no additional lexical resources) or open (no restriction). The BioCreative II gene mention task had 19 workshop participants and each team was allowed to submit up to three runs. There were no restrictions placed on the submissions. The highest achieved F score for the BioCreative I gene mention task was 0.836, while in the current challenge the highest achieved F score was 0.872. For the purpose of presenting results, and all further analysis in this paper, only the highest scoring submission (F score) from each of the 19 teams was considered.

The precision, recall, and F score for each team, in rank order based on F score, is shown in Table 1. To compute significance, bootstrap resampling was used on the test corpus. For 10,000 trials, a random sample of 5,000 sentences was selected with replacement from the test corpus, and the precision, recall, and F score was computed using these sentences for each of the 19 submissions. For each pair of submissions, say *A *and *B*, the proportion of times in these 10,000 trials that the F score of *A *exceeded the F score of *B *was noted, and we label that pair statistically significant if this proportion is greater than 95%. Significant differences are shown in Table 1. One can see that each of the three highest F scores did not have statistically significant differences. Also, each of the six highest F scores are all statistically significant compared with the remaining scores, and so on. Every pair of F scores that differed by approximately 0.0123 or more was significant, and every pair of F scores that differed by approximately 0.0035 or less was insignificant.

**Table 1:** Performance measures

Rank	*P*	*r*	F	signif	% alt
1	0.8848	0.8597	0.8721	4-19	32.48
2	0.8930	0.8449	0.8683	6-19	14.02
3	0.8493	0.8828	0.8657	6-19	14.08
4	0.8727	0.8541	0.8633	7-19	31.77
5	0.8577	0.8680	0.8628	7-19	16.67
6	0.8271	0.8932	0.8589	7-19	16.02
7	0.8697	0.8255	0.8470	8-19	14.83
8	0.8435	0.8139	0.8285	10-19	14.57
9	0.8628	0.7966	0.8284	10-19	14.55
10	0.8554	0.7683	0.8095	11-19	19.76
11	0.7295	0.8849	0.7997	13-19	16.82
12	0.9267	0.6891	0.7905	14-19	19.73
13	0.8883	0.6970	0.7811	15-19	37.05
14	0.8046	0.7361	0.7688	16-19	20.43
15	0.8228	0.7108	0.7627	17-19	16.80
16	0.8432	0.6857	0.7563	17-19	34.02
17	0.7168	0.6233	0.6668	18-19	28.23
18	0.6056	0.6411	0.6229	19	31.71
19	0.5009	0.4612	0.4802	-	28.46

The precision, recall, and F score for the best submitted run from each of 19 workshop participants, sorted by F score. Each team has an F score that has a statistically significant comparison (*P *< 0.05) with the teams indicated in the signif column. The column labeled % alt is the percentage of true positives in the submission that matched an ALTGENE annotation.

Table 1 also shows the alternates (ALTGENEs) matched in each run as a percentage of the corresponding true positives, which varies from about 15% to 30%. It is interesting to observe that the number of alternates in a run is not predictive of the score, as the top three runs represented both extremes. Nevertheless, there was an overall negative correlation of -0.40, and it could be hypothesized that methods which were less effective at learning the boundaries of the primary gene mentions were still able to get close enough to match alternatives, resulting in a higher representation of alternates among their true positives.

### Basic concepts

Before proceeding to the individual system descriptions, we give, for readers who are not familiar with natural language processing (NLP), a few paragraphs summarizing the basic terminology. For an introduction to NLP see [9] or [10]. Text is commonly processed by segmenting it into sentences or excerpts, and tokenized by breaking it up further into words, numbers, and punctuation generally called tokens, which each consist of a string of characters without white space. In this process, hyphens and punctuation often receive special treatment. A word may be further analyzed by a process called lemmatization into its lemma, which is the uninflected base form of the word that you would find as a dictionary entry. Different derivations and inflections are said to have this base form as their lemma. There is sometimes ambiguity in this concept. Alternatively, words may be stemmed by an algorithm that strips off suffixes to yield a reduced form, and this often gives a good approximation to the lemma. Tokens of text may be assigned tags which are categories from some given domain, for instance parts of speech (POS; for example, noun, verb, auxiliary). The process of identifying noun phrases and verb phrases is called chunking, which usually relies on POS tagging as its first step. As a further refinement, a sentence may be analyzed into its full syntactic structure, which is called parsing.

NER seeks to identify the words and phrases in text that reference entities in a given category, such as people, places, or companies, or in this application genes and proteins. NER is frequently accomplished with B-I-O tagging, which classifies each token as being at the beginning of the named entity (B), continuing the entity (I), or outside of any entity to be tagged (O). There are several lexical resources (sources of information about words) commonly used in solving the NER problem. A gazetteer is a list of names belonging to a particular category, such as places, persons, companies, genes, and so on. A lexicon is a source of information about different forms or grammatical properties of words. A thesaurus is a source of information indicating words with similar and/or related meanings. Systems in the BioCreative I challenge were classified as open if they used lexical resources, particularly gazetteers, and otherwise closed. A commonly used lexical resource is the Unified Medical Language System (UMLS), a controlled vocabulary of biomedical terminology maintained by the US National Library of Medicine.

Machine learning refers to computer algorithms that 'learn' to recognize concepts given a training set, which is a collection of pre-classified entities that serve as examples and counter-examples of the concept of interest. When training set examples have been classified by a human expert, the training is called supervised, otherwise it is unsupervised. Semisupervised approaches use a combination of the two. An important approach in machine learning describes each entity by a set of features, or attributes that are either present or absent for that entity. For example, the words appearing in text are frequently used as features, as are sequences of *n *words appearing consecutively, called *n*-grams. A new unseen entity can be analyzed into its description by features and categorized by a previously trained machine learning algorithm. Since most machine learning algorithms are very successful in classifying the examples of the training set, it is important to evaluate the performance of the algorithm on a test set of entities that do not appear in the training set. In this challenge, a test set was provided to participants for evaluating their systems after they were given a period of time with the training set. Often, it is necessary to divide randomly a collection (or corpus) and to use one portion as training and the remainder for testing. When this is done repeatedly it is called cross-validation. Decision trees, boosted decision trees, support vector machines (SVM), and case based reasoning are general machine learning methods. Some machine learning algorithms can be conveniently applied to problems involving tagging, including Hidden Markov models (HMM), SVMs and conditional random fields (CRFs). There are public domain libraries that are frequently used for machine learning, among them WEKA [11] for general machine learning and MALLET  for CRFs.

### Individual system descriptions

Each participating team was asked to provide a brief summary of their system for inclusion here. The contributed text is given here in the rank order of the team's best submitted run.

#### Rank 1 submission (Rie Johnson)

The focus of the IBM system was a semisupervised learning method, alternating structure optimization (ASO) [12], by which a large amount of unlabeled data (namely, unannotated Medline texts) was exploited in addition to the provided labeled training data. The experimental framework was a general purpose named entity chunking system described in [12], which uses a regularized linear classifier trained with the modified Huber loss and refers to standard features such as word strings and character types of the current and neighboring words. From these standard features, ASO creates new (and better) additional features through learning automatically created auxiliary prediction problems on the unlabeled data. The final classifiers are trained with labeled data using the standard features and the new features learned from unlabeled data. Essentially, the exploitation of unlabeled data in this manner has an effect of counteracting the unknown word problem caused by the paucity of labeled training data.

In addition to semisupervised learning, the system is equipped with optional components that perform classifier combination (combining the results of a left-to-right chunker and a right-to-left chunker similarly to the previous studies), domain lexicon lookup, automatic induction of high-order features, and simple postprocessing (parenthesis matching). The details are described in [13]. Among all the optional resources/components, unlabeled data exploited via ASO turned out to be the most effective, improving both precision and recall as well as F score by 0.0209 over the IBM base system (the system using only the standard features). The best performance 0.8721 (F score), obtained by using all the optional components, is 0.0323 higher than the IBM base system and 0.0589 higher than the participants' median.

#### Rank 2 submission (Cheng-Ju Kuo and I-Fang Chung)

Kuo and coworkers system [14], AIIAGMT, is the best performing system based on CRFs in this challenge evaluation. In fact, its performance is not statistically significantly worse than any other systems, and its performance is the best among all systems for a sample re-weighted to reflect the distribution of a random sentence extracted from Medline [5]. Its key features include a rich feature set, unification of bidirectional parsing models, a dictionary-based filtering postprocess, and its attractive high performance (especially in precision up to 0.8930 in final task evaluation). We carefully selected several feature types, including character *n*-grams (window size 2 to 4), morphological and orthographic features, but excluded some widely used features, such as stop words, prefix and suffix. Except those extensively used features, we also picked up a set of domain specific features, including abbreviations of biological chemical compounds (for instance, DNA, RNA, amino acids), compounds that co-occurred with relevant site information, and so on, for decreasing false-positives among terms with a gene mention-like morphology. Moreover, to include contextual information, we utilized -2 to 2 as the offsets to generate contextual features before any model operations.

For machine learning, we used MALLET to implement CRFs and to perform training and testing. Then, by using those features, the system under development already outperformed previous work. However, after several inside tests, we realized that the performance of a single CRF model had reached a performance plateau. Therefore, we applied a reverse data parsing (this idea became well known partly due to yet another multipurpose chunk annotator [YamCha] [15]) called 'backward parsing' to parse sentences from right to left, rather than the usual direction, to generate one more CRF model. In this way we had two divergent models, which might be expected to recognize a different set of entities from text. By combining those results, we can obtain a set of higher recall answers than the set derived from a single model. We tried different methods, such as co-training, set operations and dictionary filtering, to combine the results of bidirectional models. We found that when unifying the outputs of bidirectional models by using MALLET *n*-best option and then using a dictionary filtering post-process to filter out noise, the system obtained the highest F score. Finally, we used this system to participate in the final official task evaluation and got the second rank among 19 workshop participants (F score is 0.8683).

#### Rank 3 submission (Chun-Nan Hsu and Yu-Shi Lin)

The system of Hanshen and coworkers [16] combines two SVM models and one CRF model to achieve one of the best F scores (ranked 3rd) in BioCreative II. In fact, even the top performing system is not statistically significantly better than this system. The high performance of this combination system reinforces a well known strategy, namely that combining multiple complementary models always improves performance. Nevertheless, the component classifiers already perform very well, mostly due to the use of 'backward parsing' and the use of a large feature set. We compared two parsing directions, forward and backward, and found that backward parsing always performed better than forward parsing for both SVM and CRF models, but there is no evidential difference between the SVM models with different multiclass extensions (one versus one and one versus all).

To apply SVM to this problem, we used a sliding window to convert the problem into a supervised classifier learning problem. During the parsing, the information from the two preceding tokens and the two following tokens are used to construct a feature vector for the classifier to assign a class label to the current token. We chose YamCha to build the SVM models because it is tuned for named entity chunking tasks.

Our feature set consists of ten feature types with a total of 123,503 predicates to characterize each word. Then we applied sliding window with width 5 to extract a total of 617,515 binary features for each word. As a preprocessing step, we used the GENIA tagger  to tokenize sentences and tag POS for training and test data. We also trained a CRF model to increase the divergence of our ensemble. The CRF model was trained using MALLET with a similar set of features.

Our final step is to determine how to integrate results of the three models mentioned above to enhance recall. We applied union and intersection to combine these models. Usually, union can enhance recall because it includes more tagging results from different models, but it also degrades precision. In contrast, intersection can filter out false positives and therefore increase precision, but at the expense of recall. To take advantage of both operations but avoid pitfalls, we applied intersection to the tagging results of the two SVM models and then union with the tagging results of the CRF model as our ensemble model. The results show that this simple ensemble model remarkably enhanced recall, with precision results dropping slightly. All F score results were ranked in the top quartile.

#### Rank 4 submission (Roman Klinger and Christoph M Friedrich)

Our approach described in [17] uses a multimodel approach with two CRFs. In general, machine-learning solutions deal with a single truth. In BioCreative II the training data contain acceptable alternatives for gene and protein names next to the gold standard. The system described in [17] focuses on the use of this additional information which is ambiguous as in real world applications where different annotators build a corpus. An example from the training data is the sentence 'On the other hand factor IX activity is decreased in coumarin treatment with factor IX antigen remaining normal.' The gold standard is 'factor IX' annotated twice. The alternative annotation is 'factor IX antigen'. However, in the sentence 'The arginyl peptide bonds that are cleaved in the conversion of human factor IX to factor IXa by factor XIa were identified as Arg145-Ala146 and Arg180-Val181', the gold standard is finding 'human factor IX' and 'factor IXa' and 'factor XIa', but the alternative gives us the possibility of 'factor IX' instead of 'human factor IX'.

The set of all annotations (gold and alternatives) is split into a set of short annotations and a set of long annotations. Training the CRFs yields one system that tends to generate short annotations and one with longer annotations. The latter more often tags according to the intention of the author but has a lower recall, the first sometimes misses parts of the entity due to the characteristic of the different annotations.

To improve performance the results of the two systems are combined. Three possibilities were tested: using the result of the system with short results and adding the results of the system trained on the longer entities without overlaps, the other way round (first the long ones adding the short ones without overlaps) and combining both results with overlaps.

Training only one CRF on the information in file GENE.eval results in a lower F score than combining the results of the two CRFs by adding the short annotations to the long ones without overlaps. A remarkably high recall can be achieved by merging the results with overlaps.

The configuration of the CRFs was tuned using a 50-fold bootstrapping. It has been determined that a greedy search for the optimal feature set fails; the impact of a combination of prefixes and suffixes of length 2, 3, and 4 is much higher than expected from the impacts of the prefixes and suffixes of length 2, 3, or 4 alone. Another important feature is the use of the output of a normalizing tagger, ProMiner [18], which shows an high impact especially on the test set.

Additional postprocessing is applied, correcting frequent errors on brackets and quotation marks, as well as acronym disambiguation using latent semantic analysis.

#### Rank 5 submission (Kuzman Ganchev)

Our method [19] is similar in some respects to others that use a linear sequence model (for example, a CRF); training and test sentences are first tokenized with a rule-based tokenizer, and the goal is to assign to each token one of the three B, I, and O tags. We started with a CRF-based system similar to the one submitted by the University of Pennsylvania team to the first Biocreative competition. We made three major changes to the previous system.

• We trained the model with the *k*-best MIRA algorithm [20] using a loss function that considers alternative labelings and balances precision and recall. This allows us to trade off precision versus recall and to make use of the alternative gene mentions in addition to the gold labeling. We are in the process of releasing source code of MIRA training for use with the MALLET machine learning toolkit. The code will be publicly available at .

• We added word features based on distributional clustering of the words. An 85 million word subset of Medline was used to cluster words by bigram language model perplexity into a binary tree. Different depth tree cuts were then applied to produce five clustering features at different levels of granularity for each word type in the tree. Thus, for each word type that has been clustered there are five different non-independent cluster features generated by the clustering. On our development data, adding these features produced a 0.007 improvement in the best system and as much as 0.013 improvement in inferior systems.

• We performed feature selection by greedy search over feature templates. In the feature selection, features were grouped by feature templates. For example, there are many features for the identity of the current token (one for each token type), but we group all of these into a single 'identity' feature template. Starting with our initial list of feature templates, we repeatedly remove the one whose removal results in the greatest increase in the score on the development data, until no further improvement is possible. Removing just one feature template in this way requires training one model for each removal candidate. Once we cannot improve development data performance, we start adding feature templates from a list of candidates. This resulted in some unexpected additions and non-additions. For example, we found that adding a conjunction of four POS tags helps performance, while adding our list of gene acronyms actually hurts performance.

Even though there are hundreds of thousands of features, there are only dozens of feature templates, so doing this optimization on the development data does not lead to very severe overfitting; the F score of the final system on the development data was within 0.010 of that on unseen data. This improved performance of all systems significantly. On our development data, feature selection resulted in a 0.013 improvement in F score both when CRF and when MIRA training was used.

Together, these changes yielded an overall improvement of 0.043 in absolute performance (24% relative error reduction) over the baseline system using our development data.

#### Rank 6 submission (Manabu Torii and Hongfang Liu)

The recognition system [21] consists of three steps: name phrases in text are looked up in BioThesaurus [22] and UMLS Metathesurus [23]; a trained CRF model is applied to classify tokens in the text into three categories (B, I, and O) using lexical features and dictionary-lookup results; and postprocessing procedures are applied to correct certain errors and to make tagging results consistent. The following details the three steps.

#### Dictionary-lookup

To enhance the coverage of name phrases in dictionaries (BioThesaurus and Metathesaurus), while avoiding false positive detections during lookup, we filtered out certain types of phrases. First, phrases in BioThesaurus whose occurrences were found to be (mostly) false positives in the training corpus (for example, IL) were removed. Second, we filtered out phrases marked as nonsensical in BioThesaurus (for instance, hypothetical protein). Finally, phrases in Metathesaurus with semantic categories irrelevant for gene/protein name detection purposes were excluded. We used a flexible lookup method which ignores case differences, lexical variations, and certain punctuation symbols.

#### Machine learning

A sequence of tokens was transformed into a sequence of feature vectors for application of a machine learning method, CRF implementation of MALLET. Occurrences of gene/protein names were marked using B/I/O notation. Features considered at each token position are as follows:

• Tokens - the token at the position as well as preceding one and succeeding two tokens.

• Dictionary annotation - B/I/O annotation of the token with respect to dictionary lookup results (for example, B-Metathesaurus:aapp indicates the token is the leftmost word [B] of a phrase found in Metathesaurus, and that the phrase belongs to the UMLS category aapp [amino acid, peptide or protein]).

• POS - a part of speech tag assigned to the token by the GENIA tagger.

• Token shape - shape of the token obtained by converting each lowercase character into *a*, each uppercase character into *A*, and digit into 9 (for example, from Asp5 → Aaa9).

• Suffix - the right-most four letters of the word.

#### Postprocessing

The postprocessing procedure was implemented for the correction of apparent errors (for example, mismatching parentheses). Also, if a phrase is tagged as genes/proteins, then all of its occurrences within the same sentence should be tagged as genes/proteins consistently. Similarly, acronyms/abbreviations and their corresponding long expressions, if detected, were tagged consistently.

While the system featuring dictionary annotation outperformed one without using it in the experiments, we observed true gene/protein phrases correctly tagged during dictionary - (BioThesaurus) lookup were sometimes falsely un-tagged by the machine learning model in the final outputs. To reclaim such un-tagged phrases, two solutions were tested. The first solution was to tag different occurrences of a phrase consistently within one document (here, the corresponding abstract), not only within one sentence as already done by the postprocessing procedure. The second solution is to introduce another tagger to confirm the dictionary annotation independently of the CRF tagger. We applied the LingPipe tagger  which exploits orthographic features, and phrases tagged by both the LingPipe tagger and the BioThesaurus lookup procedure were added to the output from the CRF tagger.

#### Rank 7 submission (Barry Haddow)

To address the GM task, we employed two different machine learning methods using the same feature sets [24]. Runs 1 and 3 used CRFs with different settings of the Gaussian prior, whereas run 2 used a bidirectional maximum entropy Markov model (BMEMM) [25]. In all runs only the gold standard annotations were used in training; the alternative annotations were not used. It was found that CRF outperformed BMEMM, both using cross-validation on the training set, and on the official test set.

Before training or tagging the sentences, they were passed through a series of linguistic preprocessing stages, including tokenization, lemmatization, part of speech tagging, chunking and abbreviation detection (using the Schwartz and Hearst abbreviation detector [26]). The feature set passed to the machine learners consisted of a core set of features used in newswire named entity recognition (as in the CoNLL-2003 challenge [27], for example) augmented with extra features tailored to the biomedical domain. The core features consisted of word and part of speech tags taken from the local context, orthographic features and the head noun determined by the chunker.

The extra features consisted of both orthographic features, and features derived from the abbreviation matcher and from an in-house protein gazetteer derived from RefSeq. For the orthographic features, a set of regular expressions from the biomedical NER literature were used, with a corresponding feature recognized every time a word matched one of the regular expressions. The regular expression set represented patterns commonly found in gene or protein names.

To add the gazetteer features to each word in a given sentence, the gazetteer is first used to generate a set of matched terms for the sentence, where each word is only allowed to be in one matched term and earlier starting, longer terms take precedence. The unigram gazetteer feature for each word has value either B, I or O, depending on whether the word is at the beginning, inside, or outside of a gazetteer matched term. The bigram gazetteer feature is also added, and this is the concatenation of the previous and current word's gazetteer feature. In addition, the abbreviation feature is added to all identified abbreviations whose antecedent is found in the gazetteer.

#### Rank 8 submission (Craig A Struble and Richard J Povinelli)

Below is a brief description of a system for gene mention identification. A more complete description is contained in [28]. Our system tags a sequence of text tokens with labels indicating the location of gene/protein mentions. This is similar to gene finding algorithms that tag portions of genomic sequences with labels for gene structure, such as introns and exons.

Sentences are tokenized into numbers with optional decimals and leading signs, alphanumeric strings with single quotes (for tokens like 5'), and punctuation marks. For training and tagging, tokens are labeled with one of three labels B-GENE, I-GENE, and O representing the beginning, inside, and outside of a gene mention.

Gene mention tagging employs CRFs, a conditional probability model for tagging sequences. In most previous work with CRFs, a single linear-chain model is employed for tagging. In our system, two models were used: a first-order model in which features depend on the observation sequence and the current token label as represented by *f*_*j*_(*s*_*i*_, *o*, *i*); and a second-order model more commonly used in linear-chain CRFs in which features depend on the observation sequence, the previous token label, and current token label, as represented by *f*_*j*_(*s*_*i*-1_, *s*_*i*_, *o*, *i*).

When multiple models are used, a method for combining results is necessary. A mention was tagged if either model identified a token as being part of a gene. For overlapping tags, the starting and ending boundaries were defined by the second-order model.

Boolean features of the text were used. Orthographic features included: the token, all capital letters, all lowercase letters, punctuation, quote, alphanumeric, lower-case letters followed by capital letters, initial capital letter, single capital letter, single letter, all alphabetic, single digit, double digits, integer, real number, contains a digit, three letter amino acid code, contains globin or globulin, contains a Roman numeral, or contains a Greek letter. Additional features included prefixes and suffixes of lengths 2 to 4 and inclusion in a short form or long form of an abbreviation definition. Contextual features included features of the two preceding and following tokens.

CRFs can inadvertently label tokens as gene mentions because of orthographic similarity. It is possible to infer from the rest of the sentence that no mention exists. A character *n*-gram model was used to classify sentences into those with gene mentions and those without. Character *n*-gram models calculate the probability of class membership based on length *n *subsequence probabilities.

A postprocessing step ignored gene mentions containing mismatched parentheses, which indicated a tagging mistake.

Combining models reduced precision (0.0181) but improved recall (0.0175) and F score performance slightly (0.0008) over second-order models alone. The *n*-gram model performed surprisingly well, with a precision of 0.8724 on the test set. Using the *n*-gram classifier improved precision by 0.0303 on average, but reduced recall by 0.0420 on average, resulting in an F score reduction of 0.0078. Replacing with a better performing classifier such as support vector machines could further improve performance.

#### Rank 9 submission (Andreas Vlachos)

The main components of our system [29] are the CRF toolkit MALLET and the RASP syntactic parsing toolkit , which are both publicly available. It is worth pointing out that the system created is entirely domain independent and it could be used as it is for any NER task. The CRF models created were second order and they were trained until convergence. The features used include the token itself, whether it contains digits, letters or punctuation, information about capitalization, prefixes, and suffixes. In addition to these standard features, we extracted more features from the output of the syntactic parser for each sentence. The part of speech tagger of the RASP toolkit was parameterized to generate multiple POS tags for each token in order to mitigate unseen token errors. The syntactic parser used these sequences of POS tags to generate parses for each sentence. Its output is in the form of grammatical relations (GRs), which specify the links between the tokens in the sentence according to the syntactic parser and they are encoded in XML. From this output, for each token the following features are extracted (if possible):

• The lemma and the POS tag(s) associated with the token.

• The lemmas for the previous two and the following two tokens.

• The lemmas of the verbs of which this token is subject.

• The lemmas of the verbs of which this token is object; the lemmas of the nouns of which this token acts as modifier.

• The lemmas of the modifiers of this token.

Adding the features from the output of the syntactic parser allows the incorporation of features from a wider context than the two tokens before and after captured by the lemmas, since GRs can link tokens within a sentence independently of their proximity. Also, they result in more specific features, since the relation between two tokens is determined.

It must be mentioned that syntactic parsing is a complicated task and therefore feature extraction on its output is likely to introduce some noise. The RASP syntactic parser is domain independent but it has been developed using data from general English corpora mainly, so it is likely not to perform as well in the biomedical domain. Nevertheless, the results of the system in the BioCreative II GM task suggest that the use of syntactic parsing features improve performance from 0.7968 to 0.8284.

#### Rank 10 submission (William A Baumgartner Jr and Lawrence Hunter)

The Center for Computational Pharmacology's system for the 2006 GM task [30] focused on simple approaches for combining the output of multiple gene mention identification systems (gene taggers). We used two publicly available gene taggers, and a gene tagger developed in-house for the inaugural BioCreative GM task.

Two general strategies for combining gene tagger output were used to test two distinct hypotheses. Our first hypothesis, the 'consensus hypothesis', posited that filtering the output of multiple gene mention identification systems by requiring agreement by two or more of the individual systems would result in an overall precision measure greater than or equal to the highest precision measure of the individual systems. Our second hypothesis, the 'combining hypothesis', posited that combining the output of multiple gene mention identification systems would result in an overall recall measure greater than or equal to the highest recall measure of the individual systems.

We implemented two methods for combining the output of multiple gene taggers to test these hypotheses. To test the consensus hypothesis, we built a consensus-based filter with variable thresholds for consensus determination. This filter implements a simple voting scheme in which each tagger is given an equal vote. We then varied the consensus threshold from three (all taggers agree) to two (two of the three taggers agree). If a particular gene mention accumulates the required threshold of votes, then it is kept. If the threshold is not met, then the gene mention is not returned. By combining the votes of three taggers that each have been shown individually to have competitive performance, we expected that the consensus approach would result in an elevation in overall precision for the aggregate system, without dramatically decreasing recall.

To test the combining hypothesis, we implemented a filter that keeps all gene mentions labeled by the individual taggers. Unlike the consensus filter, this filter attempts to deal with issues of differing boundaries in the outputs of the individual taggers. When two gene mentions are found to overlap, the filter keeps the longer gene mention and discards the other. An alternative would be to keep the shorter mention; having noted that BioCreative I task 1A [3] systems (for gene mention recognition) that took steps to extend multi-word name boundaries rightward and leftward benefited from doing so, we chose to keep the longer span. By retaining all gene mentions, we expected to increase the recall of the system; however, we also expected the precision of the system to suffer, since more false positives were likely to be returned.

When evaluated against the 2006 GM task held-out test data, the results were consistent with both hypotheses. The consensus filter approaches were observed to elevate precision over any of the individual gene taggers. The overlapping filter also behaved as expected, by increasing the aggregate system's overall recall measure, with the consequence of a noticeable loss in precision. The question of the optimal number of NER systems to use for this approach remains uninvestigated. However, our findings suggest that as few as three systems are sufficient for gearing a gene mention identification system either toward maximizing precision or maximizing recall, and therefore would enable a user to fine tune a system to the task at hand.

#### Rank 11 submission (Bob Carpenter)

Alias-i submitted two systems based on our LingPipe natural language processing software [31], a first-best system and a confidence-based one. Both submissions used LingPipe out of the box without any domain-specific parameter tuning or external resources.

Both submissions are based on an underlying first-order HMM with emissions modeled by boundary-padded character language models. The chunking problem is encoded using begin/middle/end/whole tags for tokens in gene mentions and those not in gene mentions, producing an implicit second-order context coding. For example:

[BOS] p53/W-Gn regulates/W-O human/B-Gn insulin/M-Gn-/M-Gn like/M-Gn growth/M-Gn factor/M-gn II/E-Gn gene/B-O expression/M-O through/M-O active/E-O P4/B-Gn promoter/E-Gn in/B-O rhabdomyosarcoma/M-O cells/M-O./E-O [EOS].

For instance, tagging 'gene' as B-O means it is generated from a distribution of first words after a gene name. Inference in the confidence-based system is based on a generalization of the forward-backward algorithm for HMMs commonly used in speech recognition phrase detection; it uses the forward and backward estimates to the chunk's boundary along with the emission and transition probabilities for the chunk internally.

Confidence-based gene extraction, including sentence detection and input/output runs at 330,000 characters/second on a modest desktop, allowing all of MEDLINE's titles and abstracts to be analyzed in 8 hours. Recall/precision operating points for high recall were 0.95 recall at 0.18 precision, 0.99 recall at 0.11 precision, and 0.9999 recall at 0.07 precision.

Our first-best submissions involved rescoring *n*-best sequence output from the HMM decoder (Viterbi forward, exact A* backward). The rescoring model was also generative, producing entire spans with encoded boundary transitions as character language models. Full details of LingPipe's HMM rescoring model are provided in [32]. Rescoring *n*-best output is considerably slower than confidence-based gene extraction, requiring an additional 1/10,000 of a second per character to rescore 100 best outputs.

#### Rank 12 submission (Richard Tzong-Han Tsai and Hong-jie Dai)

Our IASL system, NERBio, formulates the GM task as a character-based tagging problem and employs CRFs to solve it. For this formulation, each annotated sentence was converted to the IOB2 format. Seven feature types are used: word, bracket, orthographical, part of speech, affix, character-*n*-gram, and lexicon. NERBio tackles three challenges of the GM task: excessive memory usage when using feature conjunctions, excessive unknown words, and long-distance dependency between tags.

First, NERBio can find the most effective set of feature conjunctions, thereby making better use of system memory. The selection process begins with two pools of features: the base pool, which contains all single features; and the feature conjunctions pool, containing all possible feature conjunctions. The sequential forward selection algorithm then compares all possible feature conjunctions, chooses the best, and moves it from the feature conjunctions pool to the base pool. In each subsequent iteration, it repeats this process, selecting and moving the top-scoring feature conjunction to the base pool until the F score stops increasing.

Second, to reduce the number of unknown words, NERBio normalizes all numerals in the test and training data to one. This simplifies gene names which only differ in their numerical parts. For example, interleukin-2 and interleukin-3 would both be normalized to interleukin-1. Lastly, the CRF model follows the Markov assumption that the current tag only depends on the previous tag. However, in the GM task there are many exceptions. A GM may depend on the previous or next GM, or words between these GMs. CRFs cannot identify this dependency because they only have access to the information in a limited context window. CRFs may fail if there are dependencies beyond this window. To work around this problem, we postprocess the text using global patterns composed of GM tags and surrounding words.

Pattern generation proceeds as follows; for each pair of similar sentences in the training set, we apply the Smith-Waterman local alignment algorithm to find the longest common string. During the alignment process, for each position, either of the two inputs that share the same word or GM can be counted as a match. The similarity function used in the Smith-Waterman algorithm is as follows:

$$Sim(x,y)=\{\begin{array}{cc} 1 & x=y\\ 1 & x's\;tag\;is\;B\;or\;I\;and\;y's\;tag\;is\;B\;or\;I\\ 0 & otherwise \end{array}$$

Where *x *and *y *refer to any two compared tokens from the first and second input sentences, respectively. On comparing the following two tagged sentences

chemical/O interactions/O that/O inhibit/O butyrylcholinesterase/B and/O combinations/O of/O chemicals/O that/O inhibit/O butyrylcholinesterase/B and/O

our system will extract the pattern, 'inhibit < GM > and'. Further details on the pattern generation algorithm can be found in [33] and [34].

After employing the three methods above, F score increased from 0.7864 to 0.8576 (postworkshop results) and the number of features dropped from 9,316,599 to 8,775,384. These results demonstrate that our strategies can both improve performance and maximize on valuable system memory.

#### Rank 13 submission (Feng Liu and Yifei Chen)

In the GM tagging task of BioCreative II, two SVMs and a set of postprocessing modules are proposed to compose our two-layer gene mention recognition system [35]. We chose the toolbox LIBSVM , a java/C++ library for training and using SVMs. One SVM was used for each recognition layer.

The first recognition layer is a text to gene mention layer, which takes original texts as inputs and predicts gene mention tags. The sentences in original texts are split into tokens based on spaces and punctuation. After tokenization, we use a BIO representation to chunk the gene mentions in training and test data. Then token, orthography, POS, prefix, suffix, and closed lexicon match are extracted to compose the feature set in this layer. The MedPost tagger [36] is employed to get domain specific POS tags of tokens. In the first layer, we build a closed gene mention lexicon by collecting all the terms that are annotated as gene mentions in training data. Uni-, bi-, and tri-grams of tokens starting at the current token are provided to match the lexicon entries using strict and partial matching strategies respectively. Also, the matching results are used as features of the current token. After training and prediction, the first layer offers the primary gene mention tags of input texts.

The second recognition layer is a gene mention to gene mention layer, which takes predicted gene mention tags from the first layer as inputs and outputs the final tags. In this layer, the only extracted feature of the current token is its predicted class label from previous layer. The main contribution of the second layer is to identify and correct automatically certain boundary and continuity errors made by the first layer.

Both layers employ a sliding window strategy to introduce neighboring knowledge of the current token. According to the different effects that surrounding tokens give to the current token, window sizes can be selected respectively for the different layers.

In order to improve the performance further, we developed an ensemble of postprocessing modules. The abbreviation resolution module can recover the errors caused by incorrectly mapping abbreviations to their full forms. The boundary check module can recover the boundary errors caused by our tokenization strategy and BIO representation. The name refinement module employs some rules to refine the recognized gene mentions by removing the redundancy and inconsistency.

Our resulting system achieves fairly high precision of 0.8883, which benefited from the second recognition layer and postprocessing modules. Nevertheless, our closed lexicon match induces a low recall of 0.6970. The reason is that our closed lexicon is merely constructed based on training data, which makes our system lack good generalization ability. After the competition, we improved our system by reforming the closed lexicon to alleviate this limitation. With partial matching strategy, the reformed lexicon can increase F score of the system above 0.85 [37]. As a conclusion, it is important to our recognition system to build an appropriate lexicon.

#### Rank 14 submission (Chengjie Sun)

The GM task can be cast as a sequence labeling problem [38]. In practice, we regard each word in a sentence as a token and each token is associated with a label. Each label with a form of B-C, I-C, or O indicates not only the category of a gene name but also the location of the token within the name. In this label denotation, C is the category label, and B and I are location labels, standing for the beginning of a name and inside of a name. O indicates that a token is not part of a name. For the GM task there is only one category, so we have three labels altogether: B-gene, I-gene, and O.

In our system, we utilize CRF model, which is a discriminative model and very suitable to the sequence labeling problem, to solve the GM task. Features are vital to system performance. Our feature types include orthographical features, context features, word shape features, prefix and suffix features, part of speech features, and shallow syntactic features. POS tags and shallow syntactic (chunking) tags are gotten by using the GENIA tagger. We found that chunk features can greatly improve system performance in the experiments in the JNLPBA2004 dataset .

We use the CRF tool in the MALLET toolkit to train the model on the given training data. No other resource or data are involved. We submitted two runs for the GM task in BioCreative II. The difference between them is that run2 uses stemmed tokens while run1 uses the raw tokens. To our surprise, we found that stemming is not helpful in the GM task. Our system's performance is comparable to what we got from JNLPBA2004 test data, but the performance is relatively low in BioCreative II. This is perhaps caused by the difference between the two corpora. Also, our system does not involve biomedical resources such as a dictionary or ontology, which also could decrease the system's performance.

#### Rank 15 submission (Sophia Katrenko and Pieter Adriaans)

Our team focused on applying semi-supervised techniques to extract gene mentions from the Medline abstracts [39]. Semisupervised methods have received much attention lately and have been used for different classification tasks. In some cases such techniques have been applied successfully; in others they did not improve the performance in comparison to supervised methods. Since it is relatively easy to sample text data from the Medline collection, we decided to study the impact of semi-supervised methods on the GM task. By doing so, we restricted ourselves to two methods, self-training, and co-training.

To carry out the experiments, we chose CRF as a learning method which has proven to provide the state-of-art results for the named entity recognition tasks. The feature set we used consisted of the orthographic features (digits, capital letters, and so on) and the contextual features (size of the context set to ± 2). We conducted experiments using different data samples from the Medline collection and decided to use the BioNLP dataset  as unlabeled data in our final experiments. We split the initial training set into two parts, where 9,000 sentences were used for training and 6,000 sentences for validating. All sentences were tokenized but not POS tagged. We did not use any other external resources, such as gazetteers or databases containing genes and proteins.

To investigate self-training in more detail, we carried out preliminary experiments on the subset of the Genia corpus used for the BioNLP shared task (2,000 annotated MEDLINE abstracts). Our results suggested that adding unlabeled data either does not change performance significantly or mainly contributes to recall. The best improvement we received was for the category protein which served as a motivation to apply self-training to the BioCreative II data set. The run we submitted had the following settings: number of iterations is equal to 5, number of instances added in each iteration is 100, and 1,000 MEDLINE sentences from GENIA corpus are used as a source of the unlabeled data. Labeled examples have always been sampled from the training dataset provided by the organizers of BioCreative II. In each iteration, only the most confident predictions are added. In this setting precision is much higher than recall (0.8228 versus 0.7108) and F score equals 0.7627. Interestingly, reduction of the labeled data does not significantly affect precision (in all experiments it is around 0.80). In contrast, recall can be boosted either by adding more labeled examples or by using a much larger pool of unlabeled instances.

Another method we explored is co-training. The main assumption behind this method is that two classifiers are trained using two different (but compatible) views, which are independent given the class labels. In the co-training setting, the number of iterations was set to 6. Surprisingly, self-training outperformed co-training (F score dropped to 0.7174). It has been demonstrated by Krogel and Scheffer [40] that co-training is beneficial if the dependency measure Φ^2 ^of the two classifiers is less then 10%. In our case, Φ^2 ^= 21%, which might explain why using co-training results in only slightly higher performance.

#### Rank 16 submission (Rafael Torres and Christian Blaschke)

TextDetective [41] distinguishes between functional annotations (a full name that describes the function of the gene/protein, for example 'thyrotropin releasing hormone receptor') and symbols (normally an abbreviation used as a name, such as TRHR). In the case of annotations, the morphology and the semantics of the words are highly indicative. For symbols, as the lexical aspects are frequently irrelevant, the system uses contextual information (the adjoining words that are related to genes and proteins) to detect gene names.

The system uses rules that are mostly manually created, as well as lexicons that are extracted from diverse sources (chemical or genomic databases), or obtained statistically by comparing the corpora of biological and non-biological documents (for example, extracting lists of words that frequently appear in the same context as gene names).

First, sentence boundaries are detected, and then tokens are assigned to specific classes using the rules and lexicons.

• Keyword: biologically relevant words that indicate essential features of genes and proteins, for example 'channel' or 'receptor'.

• Stop word: words that are very frequent in the corpus.

• Location: for example, 'membrane' and 'liver'.

• Type: words that often distinguish between similar names. These include numbers, combinations of letters and numbers, Greek letters, Roman numerals, and so on. This class also includes gene symbols, for example 'TNFalpha'.

• Accessory: relatively uninformative words that are found close to gene names, for example 'family' and 'subunit'.

• Bioword: other words with a biological meaning.

• Verb: a list of predefined verbs.

• Unknown: all others.

On the basis of this tokenization, candidates for gene names are selected. For example, in the case of functional annotations, the word sequence must contain at least one 'keyword'. Potential symbols are only formed by 'types'.

For gene symbols, both the local context (the words around a potential symbol) and the global context (taking into account all the occurrences of a symbol in MEDLINE) are evaluated. The local context uses a general model that distinguishes genes from nongenes. In the global context, a specific model for each potential symbol is generated which reflects how frequently a symbol is used to refer to genes or to other types of entities. This allows us to estimate the risk of tagging a symbol as a gene. The assignment of symbols, such as SCT, to a gene (secretin) has a high risk of being incorrect because of the ambiguity of the term (it can also, among others, mean 'stem cell transplant'), whereas others, such as CYP11B2, have a much lower risk.

The most relevant parameters that control the trade-off between precision and recall are as follows:

• The importance given to the risk factor. Increasing this value will enhance precision because more ambiguous symbols are rejected. If the value is decreased, then recall will take priority.

• The number of words that are analyzed in the context of a gene symbol. When this 'window' is large, recall increases because words farther away from the name will be taken into account and good words are more likely to be found. Precision will be higher when this window is small.

#### Rank 17 submission (Mariana Neves)

The system developed [42] uses the case-based reasoning foundations in which in a first step the cases are stored in a database to be further used in the classification of a new case. The system must search the base for the case most similar to the problem and the decision is given by the class of the case selected as the most similar.

The known case base is composed of words (gene mentions or not) that are present in the training documents and its function is to classify these known words when they appear in new documents. The features of the known cases are as follows: the word itself; whether it is a gene mention or not; whether the preceding word is a gene mention or not; and the frequency of the case, the number of times that the three other attributes appeared with the same values in the whole training set (cases are unique).

The unknown case base is composed by the format of the words (gene mentions or not), not of the words themselves as its function is to classify words unknown to system that may appear in a new document. The features of the unknown cases are as follows: the format of the word; whether it is a gene mention or not; whether the preceding word is a gene mention or not; and the frequency of the case, number of times that the three other attributes appeared with the same values in the whole training set.

As for the first attribute of format, each word was converted to a sequence of codes (letters) according to its characteristics. Complete words or parts of words that are present in a biological lexicon ('protein', 'gene', 'promoter') are substituted by the code W, Greek letters ('alpha', 'gamma') by G, special suffixes ('ase', 'ine') by S, upper cases by M, numbers by N, lower case letters by L, and the remaining symbols are kept in original format.

As for the classification step, for each of the words in the test set, the system first verifies its presence in the known case base. The system initially looks for a case in which the word is present but also the category of the preceding word is the same, so as to select the most similar case to the situation. If more than one case is found, then the one with higher frequency is selected and its category is the final answer of the system to the word. If an exact case is not found the system looks for a case with the opposite category of the preceding word. If a word cannot be found in the known case base, then a search in the unknown case base is performed. The word is then converted to the sequence of codes that represents its format and the search procedure is similar to the one described for the known cases.

#### Rank 18 submission (Preslav Nakov and Anna Divoli)

For BioCreative II [43], we used an extended version of our in-house gene recognition and normalization tool, originally developed for the TREC 2003 Genomics Track [44]. For our participation in the GM task, we downloaded the latest version of EntrezGene and extracted the IDs and corresponding fields likely to contain variations of gene names, for example name, official name, official symbol, alias, and description. The tools gazetteer was limited to these names, which were further filtered using WordNet in order to remove common words like 'or', 'and' and so on, which can be also gene names.

A set of normalization and expansion rules were applied in order to allow for some variations in form, including token rearrangement as well as removal of whitespaces, commas, parentheses and numerals. All possible normalizations and expansions of all known EntrezGene gene/protein names and their synonyms were generated off-line and then matched against a normalized version of the input text, giving priority to longer matches. The matches were then mapped back to the original text, and the corresponding IDs were assigned.

We made a clear separation between normalization and expansion rules, splitting the latter into two subgroups - strong rules and weak rules - according to our confidence that the resulting transformation reflects the original names/synonyms. The strong rules allow for minor changes only, for examples:

• Removal of white space (for example, 'BCL 2' → 'BCL2').

• Removal of non-alphanumeric characters (for example, 'BCL-2' → 'BCL2').

• Concatenation of numbers to the preceding token (for example, 'BCL 2' → 'BCL2').

The weak rules remove at least one alphanumeric token from the string. An example weak rule is the removal of trailing numbers, such as 'BCL 2' → 'BCL'. As another example, treating a '/' as a disjunction produces two new strings; for example, 'aspartyl/asparaginyl beta-hydroxylase' → 'aspartyl beta-hydroxylase' or 'asparaginyl beta-hydroxylase'. Another weak rule handles parenthesized expressions, removing the text before, within, and/or after the parentheses. For example, 'mitogen-activated protein (MAP) kinase' → 'mitogen-activated protein (MAP)', 'mitogen-activated protein kinase', 'MAP kinase', 'mitogen-activated protein', 'MAP', or 'kinase'.

These rules were given no priorities and were applied in parallel and recursively, trying all feasible sequences. For each resulting expanded variant, we recorded the ID of the source gene/protein/synonym and whether a weak rule was used at least once during its derivation. For a given variant, there are multiple possible IDs, some of which use strong rules only and others that use at least one weak rule. The strong variants are meant to be very accurate, while the weak ones are good for recall enhancement.

We submitted three runs:

• Run 1: no weak rules; no synonyms from the description field (F = 0.6015).

• Run 2: no weak rules; uses synonyms from the description field (F = 0.6229).

• Run 3: uses weak rules; uses synonyms from the description field (F = 0.6036).

The description field in EntrezGene often contains additional gene/protein synonyms, but it can contain other things as well, such as chemicals, organism names, and so on. Therefore, it is a good source for recall enhancement at the expense of precision.

#### Rank 19 submission (Manuel Maña and Jacinto Mata)

We can distinguish three different phases in the life cycle of a text categorization system: document indexing, classifier learning, and classifier evaluation [45]. There are a number of software libraries that provide support to the latest phases. However, document indexing is most often approached in an *ad hoc *fashion. Moreover, we believe that a framework is required to understand better the value of potential representation elements (attributes), not only in text categorization but, in general, in all of the text classification tasks [46].

When computing an attribute given a training instance, some criteria should be taken into account related to the set of examples that must be processed to obtain a value for an attribute of a single example. We propose the following types:

• Intrinsic. When computing an attribute for a given example, only information from that example is used. For example, the length of a text in text categorization.

• Contextual extrinsic. The information used is obtained from the processed example, but also from other examples that have a strong relation with it. For example, occurrence of a word in a text cited by the current text.

• Global extrinsic. The information comes from all the examples in the set. For example, occurrence of a word in the rest of the texts included in the set.

Our aim is to build a theoretical framework and a software library, JTLib, which must run part of the document indexing process, specifically the mapping of a document into a compact representation.

In the GM task we applied a simple process to build the classifier, with the aim being to get a first working version with low effort, and then concentrate on attribute analysis. During the first part of this process (get the working version) we used our JTLib library and the WEKA package [11] for the following stages:

• Document indexing. We used JTLib to develop an application that processes the training data to obtain a representation based on the selected attributes and configured into the input WEKA format (ARFF).

• Dimensionality reduction. Once the former ARFF file is generated, we used WEKA to process it aiming to find the attributes with best information gain. We used 28 attributes to characterize each instance. The following list collects the ranking of the most relevant attributes obtained from the application of information gain: frequentWords; frequentWordsInEntity; frequentWords -1; frequentWords +1; endingWords; frequentWordsInEntity -1; prev1Unigrams; frequentWordsInEntity +1; lettersAndDigits; startingWords; frequentWords -2; frequentWords +2; frequentWordsInEntity -2; prev2Unigrams; and hyphen. As we can see in this list, most of the attributes are contextual extrinsic or global extrinsic and only two of them (lettersAndDigits and hyphen) are intrinsic.

• Classifier learning. Using WEKA, we generated a set of models using different machine learning algorithms. From these classifiers, the C4.5 decision tree achieved the best results. The C4.5 algorithm allows one to make a pruned tree in a reduced time but increasing the error rate. We built two classifiers, both pruned and unpruned.

• Evaluation of text classifiers. The C4.5 unpruned achieves a slight improvement of the F score compared with the C4.5 pruned. However, the time needed to build the model of the pruned version is a 22% of the time required by the unpruned version. The classification time of the pruned algorithm is also much lower, being 6% of the time employed by C4.5 unpruned.

### Combined performance

We wished to know whether it is possible to improve on the best scores obtained in this workshop. To do this, we used machine learning to predict gene mentions using all of the submitted runs as feature data.

In order to simulate what might result if all of the methods were combined into a single system, we extracted features from the submitted runs. By holding out 25 sentences at a time, and training on the remaining 4,975 sentences, we could apply the resulting model to the held-out set and then merge all of the results to obtain a single 'fusion' run for all 5,000 sentences.

For each candidate, which is defined by a particular start and end offset within a sentence, the features described in Table 2 were generated. We used two different machine learning techniques with this feature data, boosted decision trees, and CRFs.

**Table 2:** Features for combined performance

*not*(*T*)	Team *T *did not nominate any gene mention that overlaps with this candidate.
*nom*(*T*)	Team *T *nominated a gene mention that overlaps with this candidate.
*Noms*(*T*, *S*)	Team *T *nominated a gene mention that overlaps with this candidate, and that starts before (*S *= -1), starts after (*S *= 1), or coincides with the start of this candidate (*S *= 0).
*Nome*(*T*, *E*)	Team *T *nominated a gene mention that overlaps with this candidate, and that ends before (*E *= -1), ends after (*E *= 1), or coincides with the end of this candidate (*E *= 0).
*nom*(*T*, *S*, *E*)	Team *T *nominated a gene mention with *S *and *E *as above.
*Noms*(*S*)	Some team nominated a gene mention with *S *as above.
*Nome*(*E*)	Some team nominated a gene mention with *E *as above.
*word*(*W*)	Word *W *occurs in the candidate.
*firstword*(*W*)	Word *W *is the first word of this candidate.
*lastword*(*W*)	Word *W *is the last word of this candidate.
*context*(*P*, *W*)	Word *W *at position *P *relative to this candidate. The possible values for *P *are -2,-1,1,2.

The features generated for each candidate gene mention, based on the submitted runs.

For boosted decision trees, the training set consisted of all candidates whose starting and ending offsets coincided with a nominated string from at least one team (but the starting and ending offsets need not both be nominated by the same team). Each character of a candidate was also required to overlap a nominated string from at least one team. This meant that every candidate had at least one 'nom' feature from Table 2. Each candidate was further marked as a 'positive' depending on whether it appeared exactly as a gene or alternate gene mention, and all other candidates were marked as a 'negative'. A boosted decision tree algorithm [47,48] was applied to this dataset (holding out 25 sentences at a time, as mentioned above) to learn to classify candidates as positive or negative. Each tree was allowed to have a depth of 5 and boosting was repeated 1,000 times. The induced set of decision trees was applied to the held-out set of 25 sentences to obtain gene mentions for them. Where gene mentions overlap, only the gene mention with the highest score is retained, so that the final result does not contain any overlapping gene mentions. We repeated this training using only 'nom' features, only 'word' and 'context' features, as well as using all features. The results are shown in Table 3, and the nomination features combined with words performed best with an F score of 0.9050. As this is 0.0329 greater than the highest F score obtained by an individual team, the difference is statistically significant.

**Table 3:** Combined performance results

Exp	Method	*P*	*r*	*F*	*signif*	*% alt*
A	CRF noalt, nom and word	0.9255	0.8885	0.9066	1-19, C-F	13.62
B	BDT nom and word	0.9221	0.8885	0.9050	1-19, C-F	25.67
C	BDT nom and word, top 10 teams	0.9118	0.8768	0.8940	1-19, E, F	23.37
D	BDT nom only	0.9092	0.8773	0.8929	1-19, E, F	25.42
E	BDT noalt, nom and word	0.9242	0.8165	0.8670	7-19, F	9.58
F	BDT word only	0.7165	0.6187	0.6640	18-19	37.07

The precision, recall, and F score of machine learning experiments to learn gene mentions using the data extracted from all submitted runs as features. Method column: BDT, boosted decision trees; CRF, conditional random fields; nom, all nomination features; word, words of candidate; noalt, alternate gene data not used. The column signif indicates the ranks of runs for which there was a significant difference, and the letters indicate the machine learning experiments for which there was a significant difference. The column % alt gives the percentage of alternate gene mentions among the resulting true positives.

We also used a CRF (with gaussian prior) to learn gene mentions [49]. Each sentence was tokenized and each token was marked as being positive or negative depending on whether it was part of an annotated gene (alternates were not used in this approach). The features described in Table 2 were generated for each token, in which, for the purposes of generating features, each token is treated as a candidate. By holding out 25 sentences at a time, the CRF was trained on the remaining 4,975 sentences (the gaussian prior defined in [49] was taken to be 1/2σ^2 ^= 300). The trained CRF was then applied to tag the 25 sentences, and any sequence of consecutive positive labels were combined into a single gene mention. The results from each set of 25 sentences were combined to form a single run. The result, shown in Table 3 was an F score of 0.9066. This is slightly higher than the result obtained using boosted decision trees (with nomination and word features), but the difference is not statistically significant.

A question of interest to us is whether the alternate annotations could be used in machine learning to improve performance in the gene mention task. There were some teams that did train with alternates, but the data from individual runs is not sufficient to settle the issue. Given that the boosted decision tree result, which uses alternates, is about the same as the CRF result, we might conclude that training with alternates does not make the task significantly easier. We therefore trained with boosted decision trees, marking candidates as positive only if they appear as GENE annotations (ignoring ALTGENE annotations). The result was an F score of 0.8670, which is a statistically significant difference from the result 0.9050 obtained by training in the same way with alternates positive. Training with alternates generated true positives that contained 25.67% alternates, while training without alternates generated true positives containing only 9.58% alternates.

We believed that the results from the lowest scoring teams, if used appropriately, could contribute useful information towards identifying gene mentions. To test the hypothesis, we trained with boosted decision trees using word features plus all nomination features from teams ranked 1 through 10 only. The result gave an F score of 0.8940, which is significantly lower than the 0.9050 obtained when features from teams with ranks 11 through 21 were included (this study included 2 submissions from teams that did not participate in the workshop). This confirms the importance of results from teams with lower individual performance. We note, for example, that the lowest ranking team obtained eight true positives that were not obtained by any other run.

## Conclusion

The submission data can be used as a source for exploring the consistency and accuracy of corpus annotations. There were no false positives common to all submissions, but there were two that were common to 17 submissions, for the names *GH *and *FAK*, both of which should have been annotated as true. (Correcting an erroneous false positive would result in an increase in the typical F score of about 0.0001.) There are more of these false positives with less than 17 common submissions that deserve further review.

Mentions with a high false negative rate may be clues to difficult or under-represented gene mentions. Studying these may give some guidance to future systems developers. We found 34 gene mentions that were false negatives in all 19 submissions, but all of these were correctly annotated in the corpus according to the guidelines released to participants along with the data. The 34 gene mentions fell into six different categories (mentions in bold):

• A total of ten common names appearing in generic contexts, such as: 'DDX3 and **core **colocalized in distinct spots'.

• A total of eight references to gene-associated objects, like antibodies, domains, enhancers, and binding sequences, such as this named reference to a specific gene domain: 'a splice invariant with an altered **PD **affecting its DNA specificity'.

• A total of six conjunctions or prepositions as part of gene names, such as: '...conserved in many **DNA and RNA polymerases**'.

• A total of five unusual gene names and/or unusual contexts, such as this name appearing where one would expect the name of a cell line: '10(5) **G418R **cfu/ml on NIH-3T3'.

• A total of four single-letter gene names, such as: '**B **and C1 fusions with yeast GAL4 DNA-binding and transcriptional activation domains'.

• One long gene name that did not admit any alternates: 'cDNA sequences encoding **ribulose-1,5-bisphosphate carboxylase/oxygenase (Rbu-P2 carboxylase) activase **from barley'.

As much as we would like to increase the representation of these and other 'difficult' gene mentions, it may be infeasible because it is likely that they obey a Zipf-like distribution; there are as many uniquely difficult gene mentions as there are common and easy ones.

It can be argued that the difficulty experienced by human annotators in reaching mutual agreement directly limits the performance of automated systems, and this can be influenced by the clarity of the annotation guidelines. It has been pointed out that the guidelines for annotating genes are surprisingly short and simple given the complex guidelines for annotating named entities in news wires [1]. However, a gene is a scientific concept, and it is only reasonable to rely on domain experts to recognize and annotate gene mentions. Thus, the gene annotation guidelines can be conveyed by reference to a body of knowledge shared by individuals with experience and training in molecular biology, and it is not feasible to give a complete specification for gene annotation that does not rely on this extensive background knowledge. Nevertheless, we believe that some improvement could be achieved by documenting current annotation decisions for difficult and ambiguous gene mentions.

A question of some interest is whether the higher F scores by the top performing systems in BioCreative II indicate improvement over the best systems in BioCreative I. We argue that the higher F scores do indeed indicate improvements in performance. Our bootstrap testing of the BioCreative II results suggest that a difference in F scores of approximately 0.0123 or greater is significant. However, the BioCreative I test set is a 5,000 sentence random sample from the same population of sentences from which the 5,000 sentence BioCreative II test set was taken. Now bootstrapping allows us to approximate the variation in performance by BioCreative II systems on such random 5,000 sentence samples from the parent population. Thus, we may conclude that a BioCreative II system tested on any random sample of 5,000 sentences from the parent population would be expected to produce an F score within about 0.0123 points of its score on the BioCreative II test set. In particular this implies that if we could fairly test a BioCreative II system on the BioCreative I test set the result would be within about 0.0123 points of its BioCreative II score. Note that this conclusion is valid even though we cannot fairly test BioCreative II systems on the BioCreative I test set because the latter was used in training the BioCreative II systems. Based on this reasoning and the difference between the best BioCreative II scores (0.872, 0.868) and the best BioCreative I scores (0.836, 0.826) of over 0.03, one might conclude that systems have improved. However, we do not know how BioCreative I systems would have performed on the updated version of the BioCreative I data.

Nevertheless, there are several possible reasons to believe there has been an improvement in system performance as measured in BioCreative II. First, the size of the training data is 50% greater and this would be expected to confer some improved performance, especially since there are many gene/protein entities that occur only infrequently in the data (a somewhat Zipfian behavior). Second, conditional random fields have proved to be a very effective approach to named entity recognition and were used by 11 of 19 teams in BioCreative II, while they were only used by one of the fifteen teams in BioCreative I. Third, labeling gene/protein names with a B on the first token of a name, an I attached to any subsequent token within a name, and an O attached to any token not part of a name has been an important strategy and it has led to the observation that this labeling can be done in both the forward and the backward direction in a sentence and the combined results give improved performance. This forwarded plus backward parsing was only used by the top performer in BioCreative I, but was used by the three best performers in BioCreative II plus others. Finally, more effort was put into defining effective feature sets for learning in BioCreative II and in particular the top performing system introduced a new method of using unlabelled data called alternating structural optimization which proved very effective.

The highest F score obtained in the BioCreative II evaluation was 0.8721, and we have shown that by combining the efforts of all systems it is possible to achieve an F score of 0.9066, a significant improvement. This proves that future systems should be able to achieve improved performance. We are also optimistic that, through a combination of refining the corpus for annotation consistency and improving systems design through collaboration, even greater improvements in performance are achievable.

## Abbreviations

ASO, alternating structure optimization; BMEMM, bidirectional maximum entropy Markov model; CRF, conditional random field; GM, gene mention; GN, gene normalization; GR, grammatical relation; HMM, Hidden Markov model; NER, named entity recognition; NLP, natural language processing; POS, parts of speech; PPI, protein-protein interaction; SVM, support vector machine; UMLS, Unified Medical Language System; YamCha, yet another multipurpose chunk annotator.

## Competing interests

The work of Rie Johnson was supported by IBM. The work of Roman Klinger and Christoph M Friedrich was funded in part by the Fraunhofer-Max-Planck Cooperation 'Learning and Inference Platform' and the Fraunhofer Society (a public, nonprofit research organization), which also licenses the ProMiner software. The work of Kuzman Ganchev was supported by NSF ITR EIA-0205448. The work of Barry Haddow was funded by ITI Life Sciences, Scotland, whose mission is to explore commercialization of promising technologies in the life sciences. The work of Bob Carpenter was supported by Alias-i and Grant Number R44 RR020259 from the National Center for Research Resources (the content is solely the responsibility of the author and does not necessarily represent the official views of the National Center for Research Resources or the National Institutes of Health). The work of Christian Blaschke and Rafael Torres was supported by Bioalma. The work of Manuel Maña-López and Jacinto Mata has been partially supported by the Spanish Ministry of Education and Science and the European Union from the European Regional Development Fund (ERDF) - (TIN2005-08988-C02-02). All other authors have declared that they have no competing interests.
